# Infrasound sensation is mediated by intracochlear electrical potentials

**DOI:** 10.1038/s41598-026-50179-w

**Published:** 2026-04-24

**Authors:** Carlos Jurado, Torsten Marquardt

**Affiliations:** 1 Audiology group, Department of Neuromedicine and Movement Sciences, Norwegian University of Science and Technology, Trondheim, Norway; 2 UCL Ear Institute, University College London, London, UK

**Keywords:** Biophysics, Engineering, Neuroscience, Physiology

## Abstract

Sound below 16 Hz, commonly referred to as infrasound, has no tonality and is often regarded as inaudible despite being clearly perceivable at sufficient intensity. Perception mechanisms have remained elusive. One factor decreasing hearing sensitivity towards the lowest sound frequencies is the velocity-coupled sound input to inner hair cells (IHCs), which convert cochlear mechanical vibrations into neural signals. We used non-invasive methods in humans to show that in the infrasound range, the velocity of the mechanical stimulus is reduced to such an extent that displacement-coupled outer hair cells (OHCs) start to be involved in the neural excitation, without mechanical activation of IHCs. A proposed model illustrates how OHC-generated electrical potentials act on the IHC cell membrane and so plausibly cause synaptic release that leads to auditory sensation. This mechanism explains perceptual characteristics specific to infrasound, such as the shallow slope of sensation threshold curves below 16 Hz and the abnormal growth of loudness with only small increases in sound pressure. This new physiological insight might help to understand globally spread complaints about very low-frequency environmental sounds.

**Supplementary Information:**

The online version contains supplementary material available at 10.1038/s41598-026-50179-w.

## Introduction

**Fig. 1:**
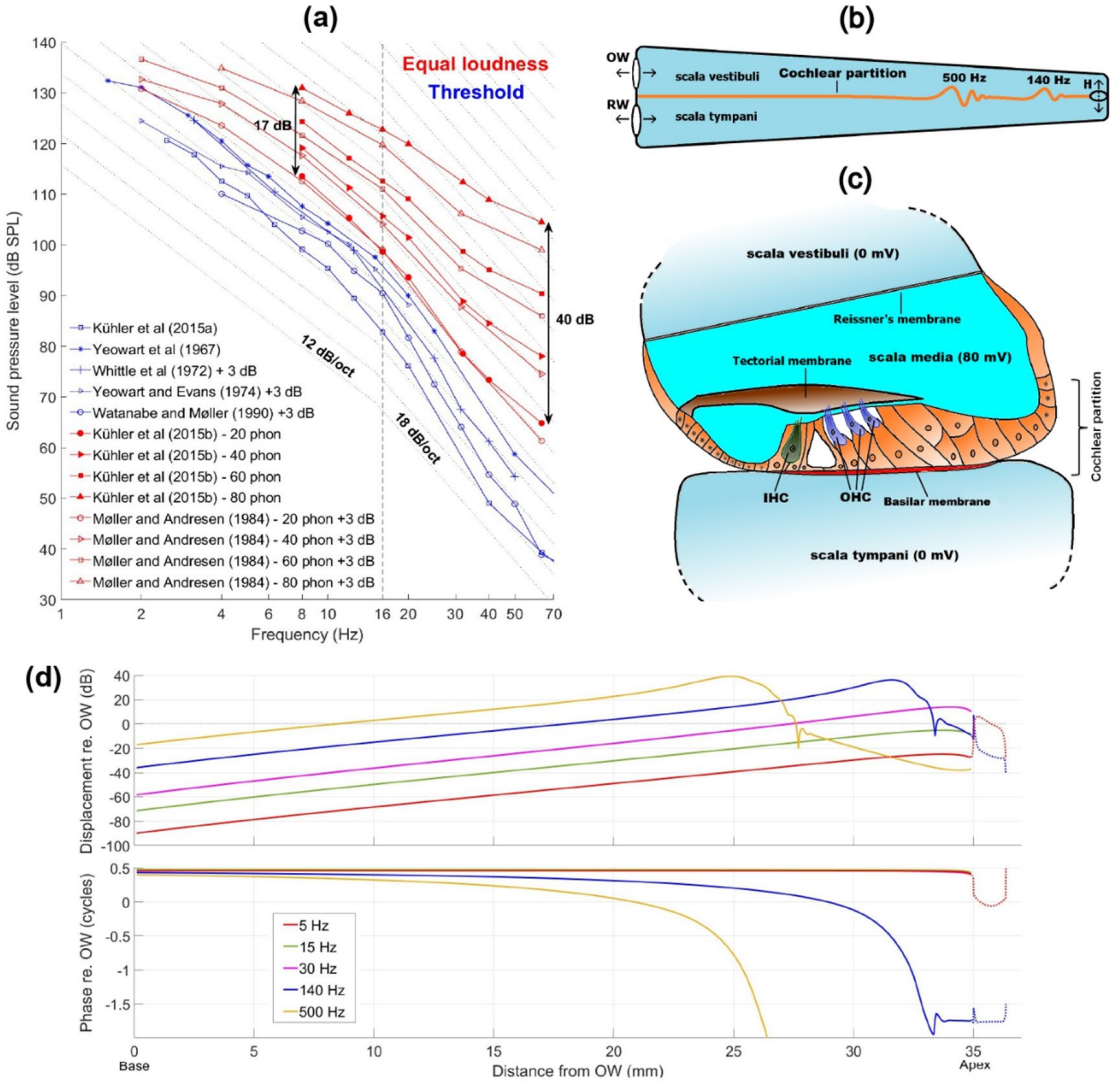
Understanding our sensitivity to low-frequency sounds and its physiological basis. (**a**), Sensation thresholds (blue) and equal-loudness contours (red; also referred to as “isophone curves”) from various literature sources spanning the infrasound and low-frequency regions^6–12^. Binaurally obtained data have been adjusted by + 3 dB to compare with monaural data that do not benefit from binaural gain^1^. For guidance, grids of 12 and 18 dB/octave are respectively shown below and above 16 Hz (vertical line). Arrows show the sound pressure level difference corresponding to a 60-phon loudness level difference at 8 Hz and 64 Hz for the dataset by Kühler and colleagues^11^. (**b**), Schematic of an uncoiled cochlea (not to scale). OW: oval window; RW: round window; H: helicotrema. Travelling waves in response to a 500-Hz tone and a 140-Hz tone were simulated with a finite-element model of a passive non-tapered cochlea with a tapered partition (for details on this model, see^13^. (**c**), Cross section through the scalae including details of the cochlear partition. Whereas the hair bundles of outer-hair cells (OHCs; blue cells) are embedded into the overlying tectorial membrane, inner-hair cells (IHCs; green cell) have free-standing hair bundles. This makes OHCs sensitive to the cochlear partition displacement and IHCs sensitive to its velocity (below 400 Hz). (**d**), Simulated magnitude and phase of the cochlear partition displacement for various stimulation frequencies. Phase is defined as partition displacement towards scala tympany relative to oval window inward displacement. For clarity, the magnitude and phase of the fluid displacement inside the helicotrema are shown for 5 Hz and 140 Hz only (dotted lines). Note how at 5-Hz stimulation, this shunts the pressure difference across the partition so that the maximum displacement on the partition is ~ 30 times smaller than that of the helicotrema fluid. Basal tail slopes of the displacements are ~ 9 dB/octave.

Sensitivity to sound decreases rather gradually as frequency approaches infrasound^1,2^. So why is human hearing conventionally thought stop below 16 Hz, and sound in this range is called *infrasound*? Has sound below 16 Hz a different perceptual quality? Tonality of sinusoidal signals starts to emerge above ~ 20 Hz^3^ whereas below ~ 12 Hz, steady tones are not heard as “continuous” but their individual cycles are perceived as being clearly segregated from one another^4^. Aside from these perceptual qualities, our current report focuses on two phenomena occurring below 16 Hz that have been quantified repeatedly with psychophysical methods but have so far remained unexplained:

(1) While our sensitivity to sound is progressively reduced with decreasing frequency, with pure-tone sensation thresholds reaching a slope of more than 18 dB/octave between 40 Hz and 16 Hz, below 16 Hz this slope decreases again to just 12 dB/octave (Fig. 1a, blue curves).

(2) At very low frequencies, the loudness of tones grows with increasing sound pressure exceptionally fast, which quickly raises their annoyance once audible^5^. The steep loudness growth here is illustrated by the higher density of curves connecting the sound pressures of tones of various frequencies that are subjectively judged as being equally loud (equal-loudness contours; see Fig. 1a, red curves).

These two psychophysical phenomena led us to the hypothesis that a different transduction mode is operational below 16 Hz where the conventional mechanical activation of cochlear sensory cells becomes ineffective, so that electrical activation dominates.

### Cochlear acoustics in response to infrasound

Much of our current knowledge about the cochlear response to low-frequency sounds is well established and has been reviewed^14,15^. Acoustically seen, the cochlea consists of two fluid-filled ducts that are separated by the compliant cochlear partition (Fig. 1b), which includes the organ of Corti with its sensory hair cells (Fig. 1c). The middle ear provides mechanical input to the oval window at the basal end of the upper duct (scala vestibuli). Below its resonance frequency, the middle ear impedance is dominated by its stiffness so that the displacement of the oval window is proportional to the sound pressure in the ear canal. The very compliant round window at the basal end of the lower duct (scala tympani) acts as a pressure release. At the apical end, both fluid ducts are connected via a small opening, the helicotrema. It prevents displacement of the partition during static oval window displacement (0 Hz) by atmospheric pressure changes.

Dynamic oval window displacement, however, creates alternating fluid pressure differences across the partition, setting off a wave along the partition that travels towards the apex. Because the compliance of the partition increases from base to apex, the wave slows down (its wavelength shortens) and its amplitude grows. Below a certain wavelength (~ 0.25 mm), a rapid increase in power dissipation hinders further growth, and the amplitude decreases sharply^16–18^. The location of the displacement maximum moves apically as the stimulation frequency is lowered. Past research has shown that below approximately 80 Hz, the wavelength remains too long at the apex to exhibit a well-defined peak^19^, and our simulations with a finite-element model (Fig. 1d) confirm that oval window stimulation below 40 Hz simply pulls and pushes the fluid through the helicotrema without an appreciable phase difference in the partition displacement along the cochlear length^13,20,21^. Nevertheless, without the formation of a travelling wave below 40 Hz, the alternating pressure difference associated with accelerating the inert fluid displaces the cochlear partition, excites the sensory cells and causes an auditory sensation. Maintaining a constant cochlear partition displacement amplitude when lowering the frequency further, requires keeping the fluid acceleration and consequently the pressure difference across the partition constant by increasing the sound pressure in the ear canal (and consequently the oval window displacement) at 12 dB/octave. In other words, the shunting effect of the helicotrema constitutes a second-order high-pass filter, which can explain the loss of cochlear sensitivity below 40 Hz^21–23^.

The reason for the even steeper threshold and equal-loudness curves above 16 Hz (Fig. 1a) lies in the mechanical activation mode of the sensory hair cells that subsequently activate their associated auditory nerve fibres: The organ of Corti (Fig. 1c) contains two types of hair cells, inner hair cells (IHCs; green cell) and outer hair cells (OHCs, blue cells). The electrical current through both types of cells is determined by the deflection of their hair bundles, mechano-sensitive collections of giant microvilli called stereocilia, arranged in a staircase pattern that when displaced trigger the opening of ion channels. The tallest stereocilia of the OHC hair bundles are attached to the overlying tectorial membrane. This direct connection means that the shear *displacement* between these cells and the tectorial membrane regulates the current into the cell soma so that a sustained OHC response is observed as long as the partition remains displaced. Their soma responds by changing their length, which regulates the sensitivity of the cochlea. The current through the IHCs, which activates the auditory nerve, is determined instead by the shear *velocity* between them and the tectorial membrane. This is because their hair bundles are not attached to the tectorial membrane so that they are deflected by the viscous flow of the sub-tectorial fluid. The effect of this additional 6-dB/octave high-pass filter due to the velocity coupling of the IHC (below ~ 400 Hz) has been experimentally shown in-vivo by numerous researchers^24–26^. This additional IHC high-pass filter, however, appears to operate only above 16 Hz, where the slope of the sensation threshold curves of at least 18 dB/octave is indicative for helicotrema shunting plus mechanical IHC coupling via fluid (Fig. 1a). Because the slope below 16 Hz “flattens” to 12 dB/octave, we hypothesize that the activation of the auditory nerve at these very low frequencies is driven by a different excitation mode that does not involve the mechanical activation of IHC. However, Dallos^27^ offered an alternative explanation of the flattening. His simple lumped-element model of low-frequency cochlear acoustics does not consider IHCs, but its helicotrema impedance has both, a mass and a viscous component. The latter changes impedance with only 6 dB/octave. Some species, e.g. guinea pigs, have relatively narrow ducts and a small helicotrema so that the viscous components dominate. This explains why in these species, the helicotrema high-pass slope is 6 dB/octave. But below a certain corner frequency, viscosity should dominate the cochlear impedance in any species because, as outlined above, the acceleration-dependent mass component declines more rapidly (12 dB/octave) than the velocity-dependent viscous component (6 dB/octave). In humans, this corner frequency might be 16 Hz so that his model could also explain the observed flattening of the threshold curves below this frequency.

**Fig. 2:**
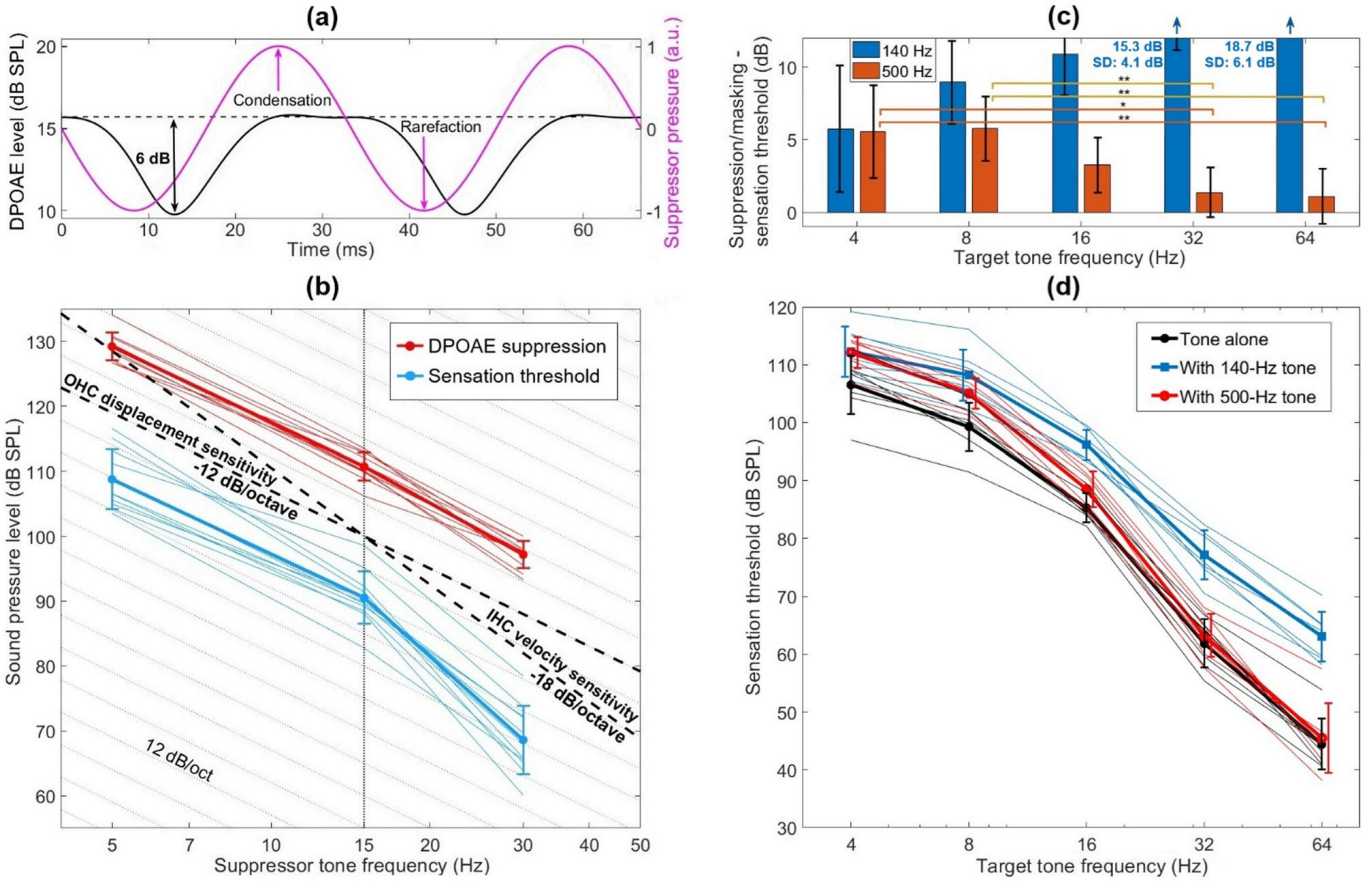
OHC sensitivity shapes the infrasound sensation threshold. (**a**), An example of a cyclic DPOAE level suppression (black solid line) as a consequence of a 30-Hz suppressor tone (magenta). Arrows indicate times of maximum (condensation) and minimum (rarefaction) pressure in the ear canal. (**b**), The required suppressor level for 6-dB suppression of OHC-generated DPOAE and sensation thresholds for the suppressor tone alone (red and blue lines, respectively). Individual and mean data are shown as thin and thick lines, respectively. The bold dashed lines illustrate how with decreasing frequency, the mechanical activation of the velocity-coupled IHC decreases with a steeper slope (18 dB/octave) than the displacement sensitivity of the OHC (12 dB/octave). The latter aligns with sensation thresholds below 15 Hz (vertical dotted line). For reference, grid lines are displayed with a slope of 12 dB/octave. (**c**), Mean difference between target tone sensation thresholds measured in the simultaneous presence of a 500-Hz or a 140-Hz tone (red and blue, respectively; both presented at 85 dB SPL) and thresholds obtained for the target tone alone (*: *p* < 0.05; **: *p* < 0.01). (**d**), Individual (thin) and mean data (bold) shown in (**c**) are given as detection threshold levels (*N* = 7; black: target tone alone). The presence of the 500-Hz tone caused an elevation of sensation thresholds specifically for infrasound tones, while the 140-Hz tone masked all five target tones. In all panels, error bars are ± 1 SD.

To challenge Dallos’ explanation, we first tested whether the shallower slopes below 16 Hz in the psychophysical data (Fig. 1a) are caused by such transition from inertia- to viscous-controlled impedance of the helicotrema. We measured within the same session the sensation thresholds for 5-Hz, 15-Hz and 30-Hz tones (*N* = 11 subjects), and also the relative displacement of the cochlear partition in response to these tones, using the same test conditions. Identical curve shapes would imply that the 6-dB/octave slope change at ~ 15 Hz in threshold can be simply explained by cochlear acoustics. The relative partition displacement as a function of the three tone frequencies was quantified non-invasively by inducing a cyclic suppression in the level of distortion-product otoacoustic emissions (DPOAEs) with each tone^20^. DPOAEs are intermodulation tones generated by the non-linear response of the healthy cochlea to two primary tones, which can be measured in the ear canal with a sensitive microphone. The low-frequency tones induce a cyclic DPOAE suppression by periodically displacing the cochlear partition away from its normal position (Fig. 2a). As, independent of suppressor frequency, an equal DPOAE suppression depth indicates an equal partition displacement amplitude^20^, the suppressor tone frequency was adjusted so that the 2f1-f2 DPOAE component was suppressed by 6 dB.

Interestingly, the slope of the threshold curve below 15 Hz (mean: −11.5 dB/oct) is practically identical and statistically indistinguishable from that of the DPOAE-suppression curve in this range (*t*-test: *T*_10_ = −0.17; *p* = 0.87; Fig. 2b). Only above 15 Hz, the slope of the threshold curve (mean: −21.9 dB/oct) was significantly steeper (*t*-test: *T*_10_ = 6.22, *p* = 9.92 × 10^− 5^), as anticipated by the velocity-coupling of the IHCs. Further, the DPOAE-suppression curve continues with practically the same slope from 5 Hz to 30 Hz (−13.5 dB/oct above and − 11.7 dB/oct below 15 Hz; no significant difference; *t*-test: *T*_10_ = 2.11, *p* = 0.061), demonstrating that the helicotrema impedance is inertia-controlled at least down to 5 Hz.

We conclude from these data that partition velocity controlling IHC transduction, becomes below 15 Hz so slow that the excitation of the auditory nerve is here dominated by an alternative mechanism of neural excitation that involves the still large deflection of the OHC hair bundles that follows partition displacement. We explored this hypothesis by combining further psychoacoustic experiments with simulations using an electric circuit model.

### Infrasound sensation is mediated by intracochlear electrical potentials

Apart from its acoustical properties, the cochlea has intricated electrical properties of uttermost importance to its function. So is the scala media charged at ~ 80 mV compared to the scala tympani and scala vestibuli (Fig. 1c). The inner and outer hair cells have a membrane potential of approximately − 40 mV and − 70 mV, respectively. This results in a large potential difference that drives the currents through the hair-cells’ mechanoelectrical transducer (MET) channels^28,29^. Displacement induced changes in the conductance of these channels modulates these potentials. Electrical recordings of endolymphatic potentials in the scala media of guinea pig cochleae by Salt and colleagues^30^ showed that infrasound tones produce extraordinarily large modulation by over 20% of the normal endolymphatic potential. A simple explanation is that this results from the synchronized opening and closing of the OHC’s MET channels along the cochlear partition, as it moves without phase difference along its entire length in response to those tones (Fig. 1d, lower panel). They further showed that the large potential modulation was strongly reduced by the presence of an intense 500-Hz tone.

We tested the possibility that in the infrasound range, these OHC-generated electrical potentials excite the auditory nerve, without mechanical stimulation of the IHC hair bundles. If so, the potential-reducing 500-Hz tone should cause an elevation of sensation thresholds exclusively below 20 Hz, but not above. In a new participant group (*N* = 7), we measured in the same ears sensation thresholds for low-frequency tones in the range 4 Hz to 64 Hz with and without the presence of a 500-Hz suppressor tone of 85 dB sound-pressure level (SPL). In addition, a 140-Hz tone at 85 dB SPL was also tried as suppressor, which due to its spectral proximity to the low-frequency tones, elevated the sensation thresholds of all probe tones (Fig. 2c&d, blue bars and lines). At 85 dB SPL, the resultant travelling wave will most likely activate the most apical nerve fibres, which are critical in sensing all the five low frequency tones and so masks them in the classical sense. The 500-Hz tone, however, led to an elevation of sensation threshold (Fig. 2c&d, red bars and lines) that was statistically significant only for the infrasound tones (4–16 Hz; a Bonferroni-adjusted significance level of 0.01 was used). The average threshold increase was 5.5 dB, 5.8 dB, 3.2 dB, 1.4dB and 1.1 dB for 4 Hz, 8 Hz, 16 Hz, 32 Hz and 64 Hz, respectively (one sample *t*-tests: 4 Hz: *T*_6_ = 4.6, *p* = 0.0038; 8 Hz: *T*_6_ = 6.88, *p* = 4.62 × 10^− 4^; 16 Hz: *T*_6_: 4.49, *p* = 0.0041; 32 Hz: *T*_6_ = 2.10, *p* = 0.080; 64 Hz: *T*_6_ = 1.52, *p* = 0.180). For the 500-Hz-tone, the travelling wave terminates well before it reaches the cochlear apex (Fig. 1d), and so caused no classical masking but likely the reduction of the intracochlear potentials that was directly observed by Salt and colleagues^30^ in guinea pigs.

### Possible mechanisms of electrical excitation

Numerous animal studies reported that neural excitation can at very low frequencies occur during partition displacement towards scala tympani, which is contrary to the accepted theory of excitatory IHC hair bundle deflection occurring during maximum partition-velocity towards scala vestibuli^31–35^. As an explanation, Sellick and colleagues^34^ postulated that OHC-generated extracellular potentials may act on the IHC cell membrane, leading to a transmembrane potential able to open the ion-channels responsible for the observed activation of auditory nerve fibres during partition displacement towards scala tympani. In fact, measurements of larger extracellular than intracellular potential modulation for stimulation < 100 Hz have been reported^26,36^. Also, a change in neural response phase with increasing tone frequency according to a transition from excitation by OHC-generated extracellular potentials to velocity-driven IHC receptor potentials has been reported^26,34^.

**Fig. 3:**
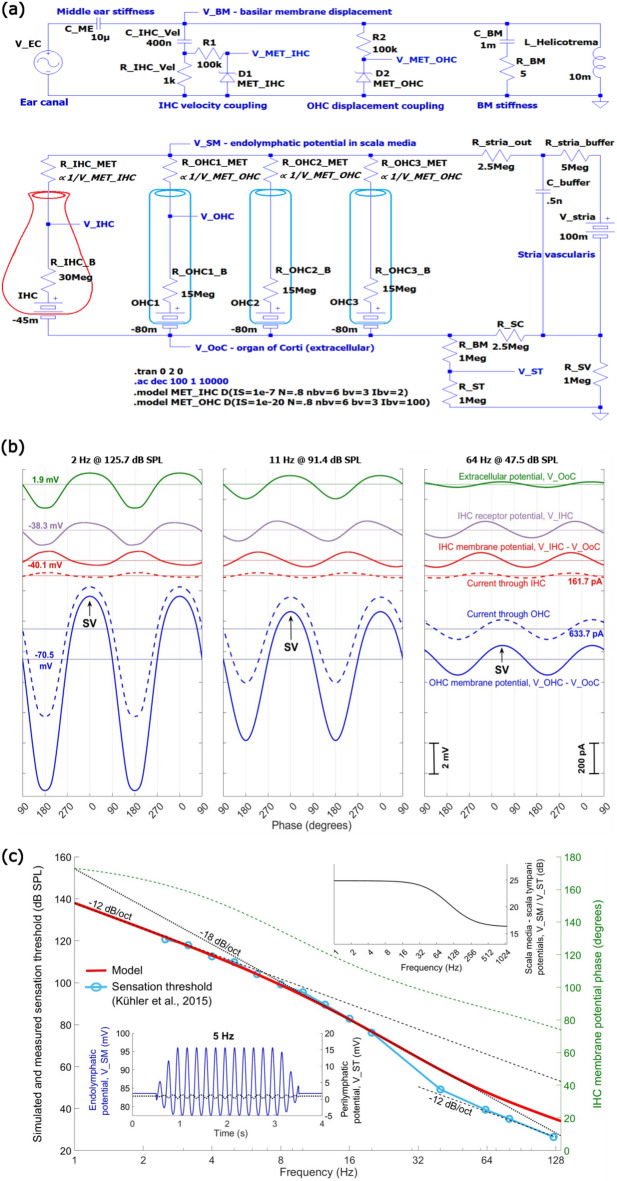
Modelling cochlear electrical excitation and sensation threshold. (**a**), Circuits of mechanoelectrical sound transduction (upper) and electrical properties within the organ of Corti (lower). (**b**), Extracellular potentials, hair cell receptor potentials, transmembrane potentials and currents through the hair cells for 2-Hz, 11-Hz and 64-Hz pure-tone stimulation at input levels producing a 1 mV_pp_ IHC transmembrane potential. Thin horizontal lines show the resting potentials/currents. Two cycles are shown, with zero phase referenced to maximum ear-canal pressure. Arrows indicate the moment of maximum partition displacement towards scala vestibuli (SV). (**c**), Model predictions (red) together with experimentally measured sensation thresholds^6^(blue). Required input levels (V_EC) were adjusted to achieve a 1 mV_pp_ iso-IHC transmembrane potential. The phase of the IHC transmembrane potential (green-dashed line) is referenced to maximum partition displacement towards SV. The lower left inset shows endolymphatic and perilymphatic potentials in response to 5-Hz stimulation at 110 dB SPL. Their frequency-dependent difference is shown in the upper-right inset. Sound pressure in dB SPL given in (**b**) and (**c**) were calculated assuming that 0-dB SPL equals V_EC = 45.6 mVpp.

We explored this possibility with an electrical lumped-element model that consisted of two circuits (Fig. 3a). The first circuit considered the mechanical properties of the middle ear, the cochlea and hair bundles and produced the variations in the conductance of the MET of IHC and OHC. This output was fed into variable MET resistances in the second circuit, that modelled the electrical cochlear properties, from which the relevant electrical potentials and currents were read (Fig. 3b). As previously postulated^34^, the simulations revealed that the two infrasound tones (2 Hz and 11 Hz) produced higher extracellular than intracellular potential modulation on the IHC membrane, resulting indeed in a depolarisation of the IHC transmembrane potential during cochlear-partition displacement towards scala tympani. In the real cochlea, this would open voltage-dependent ion-channels responsible for synaptic transmitter release and so explain the timing of the experimentally observed neural activity^31–35^. The depolarisation was the result of a current modulation through the barely mechanically-activated IHC MET, which was in opposition to that through the still mechanically-activated OHC METs, resulting in smaller potential modulations inside than outside the IHC. The current into the IHC increased during partition-displacement towards scala tympani when the closing of the OHC MET increased the across-partition potential.

The OHC transmembrane potential that resulted from the deflection of the displacement-coupled OHC hair bundles was as expected, not only in-phase with the partition movement and maximal when displaced towards scala vestibuli, but exceeded that of the IHC substantially as frequency decreased^15^. At 64-Hz stimulation, the partition velocity was large and activated the conductance of the IHC MET mechanically, which resulted in an AC receptor potential exceeding the extracellular potential modulation and so dominating the modulation of the IHC transmembrane potential (Fig. 3b). The latter was in phase with the partition velocity towards scala vestibuli as predicted by the conventionally postulated IHC mechanical activation below ~ 400 Hz^14^.

Assuming that a 1-mV_pp_ IHC transmembrane potential underlies detection threshold, we obtained the shape of the model’s detection-threshold curve and compared it to recently obtained human data^6^ (Fig. 3c). Notably, the model predicted the ~ 16-Hz transition in the sensation threshold slope from 12 dB/octave to 18 dB/octave. The flattening in the threshold slope above about 40 Hz, where the helicotrema shunt is ineffective, is also captured. Note that the experimentally obtained sensation threshold slopes in the region between 15 Hz and 40 Hz are higher than 18 dB/octave (see also Fig. 1a). One explanation for this is the weakening tonal quality and salience of tones as frequency approaches the infrasound range^3^ combined with the concurrent increase in physiological noise^3,37,38^ causing a heightening of thresholds below ~ 30 Hz. The model was also able to capture the extraordinarily large endolymphatic potentials and potential differences between scala media and scala tympani in response to infrasound reported by Salt and colleagues^30^ (lower-left and upper-right insets of Fig. 3c, respectively), both declining above ~ 50 Hz. In the model, this behaviour was realised with a capacitor (C_buffer), which stood for ion buffering inside the stria vascularis. Due to the long periodicity of tones below 50 Hz, that capacitor got periodically overcharged and drained with the closing and opening of the OHCs’ MET channels while the partition was displaced towards scala tympani and vestibuli, respectively. Above 100 Hz, the periodicity of the tones is sufficiently short for the MET conductance variation to be buffered by this capacitor so that the electrical potential variations were reduced and had hardly any impact on the mechanically driven IHC transmembrane potential.

**Fig. 4:**
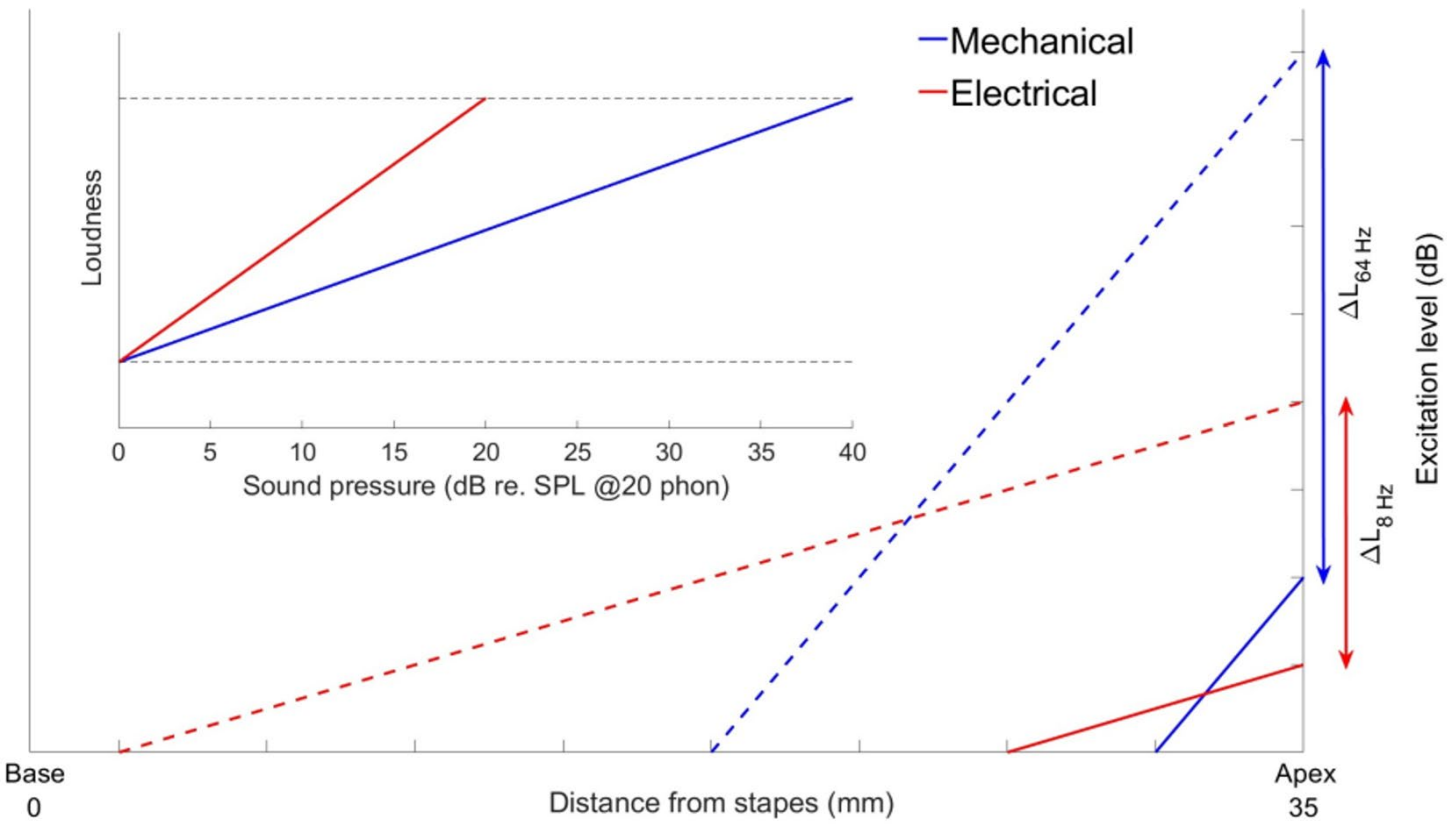
Schematic of why loudness grows faster with a shallower excitation pattern with infrasound stimulation. Here the electrical field that excites the auditory nerve fibres at 8 Hz (red) decays from its apical generation site with only a quarter of the slope than the mechanical excitation of the nerve fibres by cochlear-partition displacement at 64 Hz (blue)^13^. Loudness is estimated in models as the sum of all auditory nerve activity, i.e., it is a function of the area under the excitation pattern^39^. Starting with an equal-loudness condition by setting the smaller triangles (solid) to have equal areas (i.e., equal loudness), equal areas are maintained for the larger triangles (dashed) by increasing the 8-Hz tone level (ΔL_8Hz_) only half the amount as for 64-Hz (ΔL_64Hz_). This rough approximation fits psychophysical data surprisingly well (see vertical arrows in Fig. 1a). Conversely, loudness grows more rapidly for the infrasound tone (inset).

## Discussion

Infrasound sensation thresholds follow the slope of OHC sensitivity (Fig. 2b) and specifically increase in the presence of a spectrally distant 500-Hz tone (Fig. 2c&d) in a manner inconsistent with classical mechanical masking (Fig. 1d). Both phenomena are evidence of an electrical mode of neural excitation that operates below ~ 16 Hz. Our simulations illustrate how this excitation mode is based on variations in OHC-generated intracochlear potentials (Fig. 3b).

Threshold increases in the infrasound range caused by simultaneously presented higher frequency sounds have been reported previously^40^. It may be argued that these threshold increases and those observed here are simply the result of middle-ear muscle activation by the higher-frequency sound, that would reduce sound transmission into the cochlea. We rule out this possibility since the associated increase in middle-ear stiffness would affect transmission of all tones with frequencies below its resonance (~ 1 kHz^41,42^) and this was not observed. Also, Salt and colleagues^30^ directly measured the reduction in the electrical response by an intense 500-Hz tone with decapacitated middle-ear muscles. In line with their results, a reduction of electrical responses to a 20-Hz tone by a 1100-Hz tone has been observed also in the extracellular spaces of the organ of Corti^43^, and similar suppression effects were reported for 100-Hz IHC and OHC receptor potentials^44^. All these measurements show a larger suppression of potentials while the cochlear partition is biased towards scala tympany, a finding that cannot be explained by loss of conduction through the middle ear.

Possible intracochlear mechanisms behind this suppression remain speculative. Salt and colleagues^30^ suggested a saturation of the OHC’s MET transducer channels caused the reduction of extracellular potentials by the 500-Hz tone. However, the potentials are likely generated by OHCs located at the very apex of the cochlea, where infrasound causes largest partition displacement. Considering the steep decay of the 500-Hz travelling wave far before the apex (Fig. 1d), evidenced by a lack of masking on the 32-Hz and 64-Hz tones (Fig. 2c&d), we think that the 500-Hz tone does not saturate the MET transducer channels at these apical locations. Instead, we propose that the 500-Hz traveling wave causes an opening of the OHCs’ transducer channels at its peak location, where at threshold level, infrasound tones have an electrical impact but little or no mechanical impact. Cheatham and Dallos^43^ showed that the electrical response to a very brief 1100-Hz tone pulse in organ of Corti has a strong DC-component when presented while a 20-Hz tone displaced the partition towards scala tympany, when the potential difference across the partition is largest. We propose that this DC component is a consequence of shunting the potential difference by activation of METs located at the peak of the 1100-Hz travelling wave. A continuously presented 1100-Hz tone appears to shunt the largely apically generated 20-Hz electrical response during its entire cycle (but mostly during partition displacement towards scala tympani). Analogously, we argue, the 500-Hz tone caused a shunting of the electrical infrasound response both in our experiment and that of Salt and colleagues^30^.

### Perceptual consequences

One perceptual consequence of the electrical excitation mode is the flattening of sensation thresholds and equal-loudness contours below 16 Hz (Fig. 1a), meaning that cochlear sensitivity in the infrasound range does not decrease as rapidly with lowering frequency as it does between 16 Hz and 40 Hz. Notably, the standardized G-weighting curve for infrasound^45^, that aims to weight frequencies between 1 Hz and 20 Hz in accordance with their relative perceptual contribution, takes this flattening into consideration by having a slope of only 12 dB/octave.

A further consequence is the rapid loudness growth reported for infrasound. We showed recently^13^ that the effectiveness of an infrasound tone on suppressing the loudness of a probe tone diminishes with increasing probe frequency at a much smaller rate than expected from the mechanical displacement pattern of the cochlear partition (2–3 dB/octave versus 9 dB/octave). An explanation is that loudness suppression by the infrasound tone is not caused due to mechanically biasing the partition but due to the electrical field generated by the apical OHCs, which decays only weakly within the highly conductive cochlear fluids towards the cochlear base. If, as we propose here, OHC-generated electrical potentials can also excite the auditory nerve, the nerve activation pattern along the cochlea in response to infrasound will have the same shallow slope. Figure 4 illustrates how, with increasing sound pressure, a shallower electrical excitation slope leads to a much more rapid recruitment of basal nerve fibres than the steeper slope of mechanical displacement, resulting in a sharper increase in loudness. Because the sum of all auditory nerve activity determines loudness^39,46,^ this can readily explain that the subjectively judged loudness of an 8-Hz tone growths by a factor of 64 (a doubling every 10 phon^46^) within less than magnitude (< 20 dB) of sound pressure increase, while sound pressure increases of two magnitudes (40 dB) are required for the loudness of a 64-Hz tone to change by the same amount (see black arrows in Fig. 1a).

Environmental noises that lack spectral content above 50 Hz, lack the suppression by higher frequency sound (Fig. 2c&d) and are reported to cause considerably more annoyance and complaints than spectrally balanced noises that contain comparable loudness levels in the entire low-frequency range^47^. Already in the early seventies, Preferred Noise Criteria (PNC) curves^48^, proposed as a design goal for ambient noise in indoor environments, emphasized the importance of balanced spectral contours at low frequencies to avoid a “rumbling” sensation. Moreover, individual physiological differences in the effectiveness of higher-frequency sounds in suppressing infrasound sensation can be an explanation why only some individuals are affected by infrasound while others are barely aware of it^47,49,50^. It should be noted that evaluation of the latter hypothesis requires further dedicated experiments, which are out of the scope of the present work.

## Methods

None of the subjects participating in this study reported tinnitus, hypersensitivity to very low-frequency sounds, or a history of other hearing disorders. The absence of ear obstructions or excessive earwax were checked by otoscopy and normal middle-ear function was assessed by tympanometry. Healthy hearing status in the frequency ranges relevant to the experiments was evidenced by sufficiently strong DPOAE (i.e. the 2f1-f2 level was at least 25 dB above the local noise floor) and normal sensation thresholds (< 15 dB HL at frequencies above 20 Hz, and for the infrasound tones within 9 dB above proposed reference thresholds^1^). Data of our first experiment was collected in connection with a previous study^13^. Both experiments were approved by the UCL ethics committee (ID 0565/004) and have been performed in accordance with the declaration of Helsinki. Informed consent was obtained from all participants.

### DPOAE suppression

The sound pressure of the suppressor tone was adjusted by the experimenter so that the minimum level in the 2f1-f2 DPOAE suppression pattern was 6 dB below the unsuppressed DPOAE level. This was done for each of the three suppressor tones in the order 30 Hz, 15 Hz, and 5 Hz. The measurement order of primary pairs was randomized for each subject. The data analysis included only 12 of the 20 subjects who participated in the data collection^13^ because the 2f1-f2 DPOAE of the others could not be suppressed with the 5-Hz tone without exceeding the safety bound of 105 phon^1^. Also, one subject presented an outlier slope value, and its data was removed (see “statistics”), so that *N* = 11 for this first experiment (6 females and 5 males, 25–50 years).

Each recording lasted 20 s, was segmented into fifty 400-ms snippets, which were subsequently weighted averaged^51^ after removing noisy outlier segments^52^. Prior to these suppression measurements, the DPOAE primary parameters were adjusted for maximum emission level. The f2-primary tone level was fixed at a level of 50 phon^53^ and its frequency was restricted to a range between 600 Hz and 1000 Hz. For further details, see^20^ and^13^.

During the same session as the DPOAE suppression measurements, sensation thresholds for the three suppressor tones (2000-ms duration, including 3-cycle raised-cosine ramps) were measured for the same the ears of the same subjects. The same acoustic equipment and in-situ calibration procedure were used (see below for details).

### Infrasound desensitisation by higher-frequency tones

Sensation thresholds for five low-frequency tones (4, 8, 16, 32 and 64 Hz, with 2.5, 1.25, 1, 0.8 and 0.6 s duration, respectively; including 3 cycle raised-cosine ramps) were determined for seven subjects (4 females and 3 males, 23–50 years) with the ear of their choice under three conditions: (1) Target tone alone, (2) Target tone presented with a 140-Hz tone, (3) Target tone presented with a 500-Hz tone. The 140-Hz and 500-Hz tones were presented at 85 dB SPL in both intervals of the two-alternative forced-choice (2-AFC) detection task. They were of the same duration as the target tones and had a ramp duration of 10 ms. Measurements started always with the target-tone-only condition, followed by the two other conditions at random.

### Auditory thresholds procedure

Sensation thresholds for the low-frequency tones were measured using a 2-AFC task, with the target stimulus present in either the 1 st or the 2nd interval at random. Feedback was provided after each response. The target stimulus started at a level of 40 phon^1^^[,53^, and got reduced in 3-dB steps after each correct identification, until the subjects indicated with a special button that the level was close to their individual threshold. Then, a 3-down 1-up rule started with a 2-dB step size. After two reversals, the procedure continued for another eight reversals in 1-dB steps and the average level of these eight reversals determined the threshold. Measurements were made three times, and the order of tone frequencies to measure was randomized each time. The three thresholds were then averaged, unless one value differed from any other by more than 4 dB, in which case it was excluded from the average.

### Apparatus

Experiments were carried out at triple-walled soundproof booths of the UCL Ear Institute. All stimuli were digitally created, and the procedures controlled using custom made scripts in MATLAB (Version R2017a, The MathWorks Inc., MA, USA). Stimuli were D/A converted using a Fireface UC audio device (RME Audio AG, Germany; sampling rate: 48 kHz). The primary tones (f1 and f2) were produced by an OAE probe (ER-10 C, Etymotic Research Inc., IL, USA; this probe also housed the in-ear microphone), directly driven by an output of the audio device. In the first experiment (Fig. 2a&b), the electrical signal for the low-frequency tones was amplified (Blueprint A150; Beyerdynamic, Germany) and fed to a DT-48 headphone speaker (Beyerdynamic GmbH & Co. KG, Germany). The latter delivered the low-frequency tones acoustically via a narrow flexible tube (200 mm in length, 0.5 mm in diameter), which was pierced through the foam eartip (ER10C-14 A) of the ER-10 C before placing it airtight into the subject’s ear canal. The thin delivery tube constitutes an acoustic low-pass filter, which together with an RC low-pass filter and an attenuator (inserted between the audio device and power amplifier), prevented accidental sound delivery above 105 phon.

In the second experiment (Fig. 2c&d), the DT-48 headphone speaker was replaced by a custom-made infrasound source with very low-harmonic distortions^6^ that was located outside the sound booth. It consisted of a 15-inch dynamic subwoofer speaker (15P80Nd, Beyma, Valencia, Spain), mounted in a wooden enclosure and connected via an 8-m long polythene tube to the pressure-sealed earplug of the ER-10 C ear probe. To ensure participant’s safety, a RC-lowpass filter was inserted between the power amplifier (BAA 120, BEAK electronic, Frankenblick, Germany) and the speaker so that stimuli could not exceed ∼95 phon at maximum voltage output. The 140/500-Hz masker was produced by one of the speakers of the ER-10 C probe.

### Calibration

The microphone of the ER-10 C probe was calibrated in a 1.5-cm^3^ cavity with a B&K 4192 reference microphone connected to a B&K 2636 measurement amplifier, using its rear output (Brüel & Kjær A/S, Denmark). After each placing of the probe, the sound sources were calibrated in-situ so that the desired SPLs were measured at the probe’s calibrated microphone inside the ear canal.

### Statistics

All *t*-tests reported were two-tailed *t*-tests. When comparing two groups, these were paired-sample *t*-tests, and when applied to a single group, i.e., assessing whether their mean was equal to zero or not, these were one-sample *t*-tests. In a multiple comparison made with *n* = 5, a Bonferroni-adjusted significance level of 0.05/5 = 0.01 was used and original *p*-values are reported. Analysis of outlier slope values in the DPOAE-suppression curves was done using the interquartile range method^54^. An outlier was defined as a value below Q_1_−1.5×IQR or above Q_3_ + 1.5×IQR (where Q_1_: 1 st quartile; Q_3_: 3rd quartile; IQR: interquartile range). The method was applied separately for slopes below and above 15 Hz. For the one subject that was discarded because of an outlier slope value in its DPOAE suppression curve, its auditory threshold data was also excluded.

### Lumped-element circuit model

The model was implemented using the freely available SPICE simulator software LTSpice^55^. Its mechano-acoustical circuit was based on Marquardt and Hensel^23^. The circuit describing the electrical cochlear properties was based on that by Dallos^28^, extended by Mountain^29^. Reactive elements for the hair cells were omitted since they are irrelevant for stimulation below 500 Hz.

The MET transfer functions of IHC and OHC (Fig. S1) were realized with the custom-designed Zener diodes, D1 and D2, respectively. Their Spice directives are given in Fig. 3a. Because radially (excitatory) hair-bundle deflection is represented by a positive input, V_EC is defined as being positive during rarefaction in the ear canal. The output of the transfer functions represents the electrical conductance of the METs and are applied in the definitions of the variable resistances representing the IHC and OHC METs in the lower electrical circuit. The following equations were used:

1. a $${\text{IHC MET}}:{\mathrm{R}} = {\mathrm{15}}0{\mathrm{Meg}}/\left( {{\mathrm{2}}.0*{\mathrm{V}}\_{\mathrm{MET}}\_{\mathrm{IHC}}} \right) + 0.{\mathrm{2V}})$$

1. b $${\text{OHC MET}}:{\mathrm{R}} = {\mathrm{275Meg}}/\left( {{\mathrm{1}}.{\mathrm{7}}*{\mathrm{V}}\_{\mathrm{MET}}\_{\mathrm{OHC}}} \right) + {\mathrm{1}}.{\mathrm{2V}})$$

A crucial feature of the model is the capacitor, C-buffer, which represents an ion buffer in the stria vascularis that produces the endolymphatic potential inside the scala media. Together with its adjacent resistors, it provides the low-pass filter effect (Fig. 3c, upper-right inset) observed experimentally in guinea pigs^30^. With tones below 32 Hz, the buffer got periodically drained and over-filled with the synchronised opening and closing of the OHC METs, producing the extraordinarily large endolymphatic potential modulation (Fig. 3c, lower-left inset) as observed in-vivo^30^. At higher frequency, the periodicity was short enough to buffer the alternation in the demand of electrical charges. Note that the resting current through the two stria-internal resistors^56^ produced a drop of the stria’s internal voltage supply (V_stria = 100 mV) to the experimentally measured endolymphatic resting potential^30^ of 84 mV.

Model parameters were tuned considering values and electrophysiological recordings reported in the literature^14,20,23,26,28,30,36,43,44^.

## Supplementary Information

Below is the link to the electronic supplementary material.

Supplementary Material 1

Supplementary Material 2

## Data Availability

The data that support the findings of this study are available from the corresponding author upon reasonable request.

## Code availability

Scripts designed for data collection and analysis are available from the corresponding author upon reasonable request. The LTSpice circuit is provided as supplementary file.
