# Supplementary material for: Infrasound sensation is mediated by intracochlear electrical potentials
Source: Sci Rep. 2026 Apr 24;16:19097. doi: 10.1038/s41598-026-50179-w (PMC13280377; doi:10.1038/s41598-026-50179-w)
Supplement: Supplementary file 2 — Supplementary Material 2 [file 41598_2026_50179_MOESM2_ESM.docx]

**
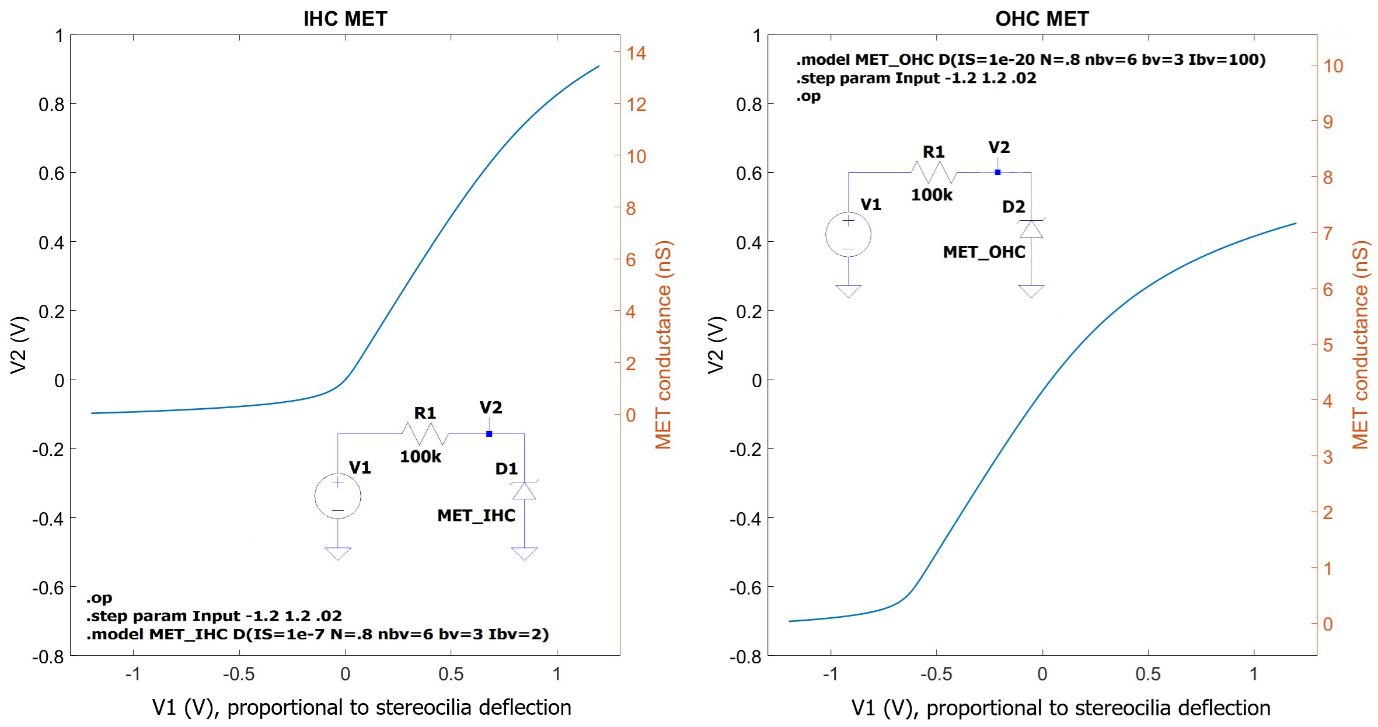
**

**Fig. S1| Simulated MET transfer functions for the IHC and OHC.** Insets in each panel show the testing circuit with the respective LTspice directives. Using equations (1.a) and (1.b), the voltage V2 (V_MET_IHC or V_MET_OHC in Fig. 3a) can be directly converted to MET conductance (right-hand-side scales). Voltage V1 represents and is proportional to the stereocilia deflection. The conversion between the two is undefined because the model does not quantify displacements.
